# Lessons Pharmacy Practice in India Should Adopt From Advanced Nations: A Review

**DOI:** 10.7759/cureus.67413

**Published:** 2024-08-21

**Authors:** Nandhini Subramaniam, Ian Osoro, Muhasaparur G Rajanandh

**Affiliations:** 1 Pharmacy Practice, SRM College of Pharmacy, SRM Institute of Science and Technology, Kattankulathur, IND; 2 Pharmacy Practice, SRM Institute of Science and Technology, Kattankulathur, IND

**Keywords:** india, board certification, specialization, clinical pharmacist, patient care

## Abstract

In India, pharmacy practice is still at a developing stage with the majority of the graduates taking the industry pathway. Currently, there are only a few pharmacists who have been board-certified by the Board of Pharmacy Specialities (BPS), which is the most established pharmacist board certification program globally. Even though India is the largest global exporter of generic medications, pharmacy practice is yet to gain stronghold within its healthcare scenarios. In this article, we aim to examine the development of pharmacy practice from a global viewpoint and scale down to the recent modern practice, particularly in advanced nations. Furthermore, we assess the ways through which pharmacy practice can be enhanced in India.

Notably, with several pharmacy practice graduates completing their studies in India yearly, pharmacy practice is projected to significantly grow in the coming years. Gaining a proper understanding of and embracing advanced clinical pharmacy practices will improve the domain of pharmacy practice among both junior and senior pharmacists. Moreover, enrolling in and receiving international accreditations such as the Board of Pharmacy Specializations (BPS) will validate the practice standards being offered in India as compared to other developed countries, i.e., the US. The main objective of this review is to assess various means through which pharmacy practice can be improved in India.

## Introduction and background

Since the early 1900s, pharmacists have performed the function of an apothecary, i.e., manufacturing medication items secundum artem (in accordance with the art) for therapeutic use [1]. However, community pharmacies faced a crisis in the 1920s as a result of the shift from compounding to premanufactured proprietary products [1]. The traditional role of the pharmacy was diminishing, and it was unclear what a pharmacist's responsibilities were if they were not compounding. While it played a significant role in the pharmacy's identity, prescription dispensing was not given as much attention as it once received. This eventually changed when pharmaceutical research advances led to a sharp rise in the number of newly developed prescription medication items in the mid-20th century [2].

By the 1950s, pharmacists' only responsibilities were to compound, dispense, and label prefabricated goods due to the pharmaceutical industry's large-scale production of medical products and the majority of therapeutic agents' new legal position as prescription only. The number of pharmaceuticals prescribed increased by more than 50% in the 1950s due to a boom of newly discovered medications [1]. In response, pharmacists established the idea of clinical pharmacy by the middle of the 1960s and changed their profession to one that was more patient-oriented. Clinical pharmaceutical services also started to emerge in the 1960s [1]. Also for American pharmacy, the 1950s through 1970s were an extremely important phase [2]. In 1969, the American Public Health Association Code of Ethics allowed pharmacists to participate in activities that might have been deemed unethical under earlier practices. Ethical standards also changed over time. These changes laid the groundwork for further developments in the 1980s, which would strengthen the belief that community pharmacists had a duty to their patients that went beyond just filling prescriptions [2].

In 1990, prescription counseling became mandatory under the federal government's Medicaid program. By the end of the 1990s, the pharmaceutical care services business had sufficiently matured [2]. In 2001, additional prescriptions by pharmacists were authorized in the United Kingdom [1]. By 2003, pharmacists were permitted to provide vaccinations in 34 states; nonetheless, their scope of practice was severely limited in relation to vaccinations. In 2004, about 5-10% of community pharmacists stated that medication therapy treatments were offered by their pharmacy. From 2009 to now, the proportion of licensed pharmacists who are unemployed has increased by twofold [2].

In the post-pharmaceutical care era, the pharmacist's role in the care of patients and non-dispensing activities has increased in the modern age, building on their emergence in earlier decades. A pharmacist's daily tasks are beginning to include more and more patient counseling, vaccinations, and other duties [3]. The secret to enabling the pharmacy practice to develop and increase its patient care role is to put a strong emphasis on collaborating with healthcare providers. Although there are many opportunities for pharmacists to take on more responsibilities in the post-pharmaceutical care era, difficulties still exist. A lot of pharmacists still encounter obstacles when trying to fully integrate patient care services due to structural issues in the healthcare industry, namely under-supportive compensation models for non-dispensing activities. Positive trends, however, point to a rising appreciation of the value that pharmacists bring to healthcare, indicating that in the years to come, their positions will likely continue to develop and grow [2].

As society changes throughout the next century, community pharmacy practice will also adapt to meet the needs of the patients. These advances will give community pharmacists more chances to offer patient care services unrelated to dispensing.

## Review

Important systems that have enhanced pharmacy practice

The practice of pharmacy has been greatly improved by several methods and technology, which have also improved patient care, efficiency, and overall pharmacy administration. Below are a few noteworthy instances:

Software for Pharmacy Management

Pharmacy management systems simplify many operations, including filling prescriptions, managing inventories, managing patient information, and distributing medications. Among the best systems are: McKesson (All-inclusive administration for pharmacies in the health system), Cerner (Unifies patient information on several platforms), and PioneerRX (Improves patient care and pharmacy profitability) [3].

Technologies for Digital Health

Digital health tools provide real-time patient interaction and data insights. Among the innovations are: Medication dispensers for use at home (gadgets like the Spencer device remind users to take their prescriptions and enable remote consultations with pharmacists) and telepharmacy: This improves access to care by allowing pharmacists to manage prescriptions and give consultations from a distance [4].

Mechanized Dispensing Devices

Robotics and automated technologies help pharmacists fill prescriptions accurately, reduce errors, and focus on patient care. Among these tools is Central Pharmacy Manager, which unifies pharmacy functions throughout various care environments, i.e., ScriptPro: automates inventory control and prescription dispensing [5].

Electronic Medication Administration Record (eMAR)

By tracking the administration of medications in real time, eMAR systems guarantee precise dosage and adherence. Healthcare and long-term care environments benefit greatly from these systems [3].

Models of Integrated Specialty Pharmacy

Patients and providers are more satisfied with these models because they centralize prior authorizations, include therapy monitoring in clinics, and provide health coaching [5].

Global pharmacy practice scenario

The United States is frequently regarded as the top nation in pharmacy practice as it offers a sophisticated healthcare system that provides a wide range of career options for pharmacists in neighborhood pharmacies, hospitals, research facilities, and academic organizations [6].

Recent decades have seen substantial changes in clinical pharmacy in America, as more pharmacists pursue postgraduate training. These days, clinical pharmacists are acknowledged for playing a critical role in patient care, especially when it comes to interprofessional collaboration, medication management, and patient education. However, a variety of social, cultural, and economic variables have impacted the region's success in this area, resulting in varying degrees of advancement among the various nations [7]. Pharmacists in Scotland, the Netherlands, Canada, England, and Australia have made significant strides toward enhancing the responsibilities of community-based pharmacists.

Medication optimization services might include services like arranging for last-minute refills, extending or renewing prescriptions, modifying the amount or formulation of drugs, and implementing therapeutic substitutions. Additionally, community-based pharmacists in Canada and the UK provide comprehensive minor illness treatment. A new funding mechanism presents an opportunity for New Zealand to increase the scope of community-based pharmacy services. Prescriptive authority will be added to South African pharmacists' qualifications, which will make it easier for community-based pharmaceutical services to grow [8]. While other Middle Eastern nations are still having difficulty expanding their practices, the UAE is taking steps to facilitate the growth of the community pharmacist's role. Although the position of the clinical pharmacist has not expanded in Asia, it has taken strides with the introduction of drug use reviews, health promotion, and health assessment [8].

There is a need to expand pharmacy services to enhance medication utilization and availability within a collaborative primary care team. This can be achieved through integrating digital health solutions into practice, showing leadership in personalized medicine, promoting wellness, preventive measures, and education to reduce the impact of chronic diseases as well as identify public health risks [7]. Furthermore, providing innovative and practical services to minimize such risks is essential. Community pharmacists rank as the third largest cohort of healthcare professionals globally, following doctors and nurses [7]. Table 1 summarizes some of the criteria for practice in these countries.

**Table 1:** Comparison of key aspects of pharmacy practice in developed nations FPGEE: Foreign Pharmacy Graduate Equivalency Examination; NAPLEX: North American Pharmacist Licensure Examination; TOEFL: Test of English as a Foreign Language; iBT: Internet-based test; PEBC: Pharmacy Examining Board of Canada; MCQs: Multiple Choice Questions; SBAQs: Single Best Answer Questions; OSCE: Objective Structured Clinical Examination; GPhC: General Pharmaceutical Council; KAPS: Knowledge Assessment of Pharmaceutical Sciences; PSI: Pharmaceutical Society of Ireland

Country	Exam	Qualification	Format of the exam	Price charged for the exam	Key aspect
USA	FPGEE, NAPLEX	Primary pharmacy qualification TOEFL iBT	Computer-based examination	FPGEE application: $90; exam: $800; NAPLEX fees: $485	Only 5 attempts are allowed for FPGEE. Only 5 attempts are allowed for NAPLEX
Canada	PEBC	Pharmacy qualification	180 MCQs in the form of SBA-Qs/3hrs	QE1: 400CAD; QE2: 1520CAD	To complete pharmacist qualifying examination part 1 & 2, i.e: part1: MCQ, part2: OSCE
UK	GPhC	M.Pharm degree from a recognized university, and then a year-long basic education program	The evaluation is computer-based, with a pause in between sections	£182	Exam: Part 1 & 2; for a candidate to succeed as a pharmacist in Britain, they must pass both exams
Australia	KAPS	A minimum of a four-year, full-time B. Pharm degree, or its equivalent, is required of candidates	A computer-based online assessment called, KAPS	AU $2290	The KAPS exam demands applicants to rely entirely on their understanding and expertise of pharmaceutical science during the test, in contrast to many other licensure exams that permit candidates to consult textbooks or notes
Ireland	PSI Equivalent	The applicant must hold a continuous five-year pharmacy degree. Accredited as a pharmacist in their native nation	Any location may be used to take the computer-proctored exam as long as the testing facility satisfies PSI standards	EUR 1,500	The fact that it can be taken online is a noteworthy advantage. Examinees can do it whenever is most convenient for them

Indian pharmacy practice scenario

India happens to be the global leading exporter of generic medicines which are easily affordable to the majority of patients, unlike branded medicines. Boasting a population of 1.4 billion people, India is termed the “Pharmacy of the world” due to its ability to consistently produce and supply cost-effective medications [8]. However, the concept of Pharmacy Practice has suffered greatly at the expense of the growing pharmaceutical industry sector. Currently, a Masters in Pharmacy Practice (two years) or a Doctor of Pharmacy (six years) courses signify an inclination towards clinical pharmacy where the number of seats is usually 15 and 30 respectively in most pharmacy institutions [9].

India has yet to embrace the role of a specialized pharmacist despite being a global market for exporting drugs. In India, community and industrial pharmacy are the common areas of pharmacy usually followed by most graduating pharmacy students. Both bachelors and masters of pharmacy programs in India tend to focus only on fundamental sciences thereby causing more graduates to be inclined towards the industry sector. Both of these two domains have a more defined role (production and supply of medication respectively) unlike clinical pharmacy which is still growing [10]. Hence, we decided to delve into a major component of pharmacy practice i.e. clinical pharmacist specialization which is aimed at improving the standards of care.

Board certification or specialization is a common practice particularly in the medical field to verify the advancement of a healthcare professional in a particular area. The US has been at the forefront of pharmacist specialization with the introduction of the Board of Pharmacy Specialities (BPS) in 1976. The BPS is a certification course that enhances the competence of pharmacists by enabling them to gain evidence-based knowledge and skills needed within a specific healthcare setting. There are 14 specialties offered by the BPS and some examples are oncology, geriatrics, pharmacotherapy, cardiology, critical care, and infectious diseases among others. Currently, pharmacist board certification is not a very popular route clinical pharmacists take in India which can be seen from the smaller number of board-certified pharmacists - four. With more pharmacy students graduating, themes such as pharmacist specialization must be emphasized among the fresh graduates who will soon become the face of Pharmacy Practice in India.

Proper medication use is among the WHO Global Patient Safety Challenge 2017 goals. With India being the leading country in the global population (1.4 billion), the healthcare needs of the citizens must be adequately met [11]. For an individual to conduct pharmacy-related services, they need to have qualified with one of the following degrees: Diploma in Pharmacy (D. Pharm), Bachelor of Pharmacy (B. Pharm), Master of Pharmacy (M. Pharm), Doctor of Pharmacy (PharmD) or Doctor of Philosophy in Pharmacy (PhD). Focusing on the young and upcoming talents in pharmacy graduates may prove to be a very good option for creating awareness and enhancing pharmacist board certification.

The D. Pharm program is usually a two-year course and those graduates intending to have a bachelor’s degree need to undergo an additional three years of training in B. Pharm. Bachelor, Master, PharmD, and PhD courses last four, two, six, and three years respectively, and the Pharmacy Council of India (PCI) and the All-India Council for Technical Education (AICTE) are the two main bodies governing the pharmacy curriculum in India [12,13]. Delays in the implementation of necessary regulations by the PCI have led to undefined duties and responsibilities of PharmD graduates although this has been currently resolved [14,15].

In 2015, the PCI clarified that pharmacy centers were not permitted to ‘diagnose disease and prescribe medicines. This meant that the PharmD graduates would have minimum contact with patients, which led to widespread protests, and in 2019, the PCI acknowledged the graduates as clinical pharmacists through an amendment that stated that they may participate in improving patient medication therapy in conjunction with physicians and other associated healthcare workers [16]. However, it was not until 2021 that the roles and responsibilities of PharmD graduates within a hospital setting were clearly outlined [17]. The Clinical Pharmacy Council is the autonomous body in India in charge of certifying and acknowledging specialized clinical pharmacists. Although this certification program has both lower stringent qualification requirements and costs, it has yet to grow and have an impact on an international level as compared to more advanced programs such as the BPS.

Factors motivating pharmacist specialization

Despite the state of pharmacy practice in India, we believe that the addition of a few more components into the practice will greatly improve its growth as a field. Patient care is the core of pharmacy practice and therefore, clinical pharmacists should be encouraged to specialize since they aim to ensure better patient outcomes. 

**Figure 1:**
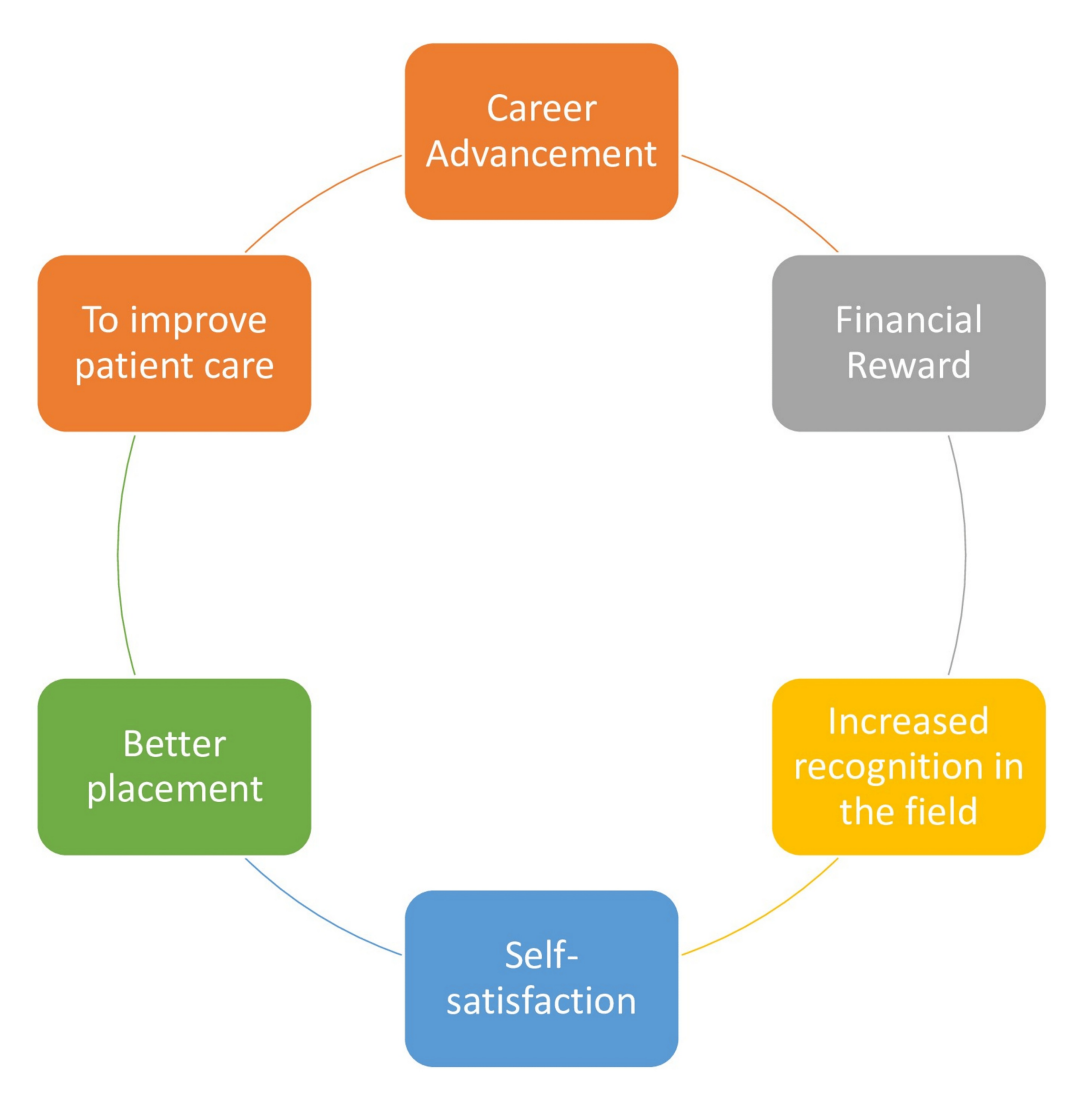
Motivating factors for pharmacist board certification This figure was created by the authors

For instance, giving better placement and good financial rewards for the specialized pharmacists within hospital settings along with the system will help improve the career prospects of pharmacy practice. Having a well-paying job with the corresponding compensation and recognition will highly elevate the scope of the job. Since we are interested in graduate students, we believe that these practices will strengthen their desire to specialize in their area of interest. Moreover, having clinical-based institutions that value specializations will significantly enhance the desire for many pharmacists to take certification exams. This is because such institutions will ensure there is room for career development, and self-improvement in knowledge and skills thereby drawing the attention of specialized pharmacists. A summary of the included factors is given below in Figure 1. 

Barriers affecting pharmacist specialization

On the other hand, it is also important to take note of what stands as a hindrance to the growth of pharmacist specialization in India. Firstly, there is little awareness about board specialization since few pharmacy graduates go into the clinical sector and those who are in this sector haven’t yet fully delved into it due to lack of opportunities among other factors. Understandably, pharmacy practice is yet at its infancy stages therefore more time has to be given for its roots to spread far in the presence of the right conditions and policies.

Additionally, we note that there is minimal to no guidance regarding pharmacist specialization. This is because even the senior clinical pharmacists haven’t gone to the extent of certification and thereby the younger ones lack the necessary guidance needed. Furthermore, the lack of financial support is a hindrance to pharmacist specialization especially in low- and middle-income countries. The current examination fee for BPS certification is $600 (₹49,859.43 INR) and $300 for those who are retaking the exams. An additional $125 is also required annually to maintain good standing with the board. Cumulatively, this amount is expensive in India and not all intending clinical pharmacists can pay it.

**Figure 2:**
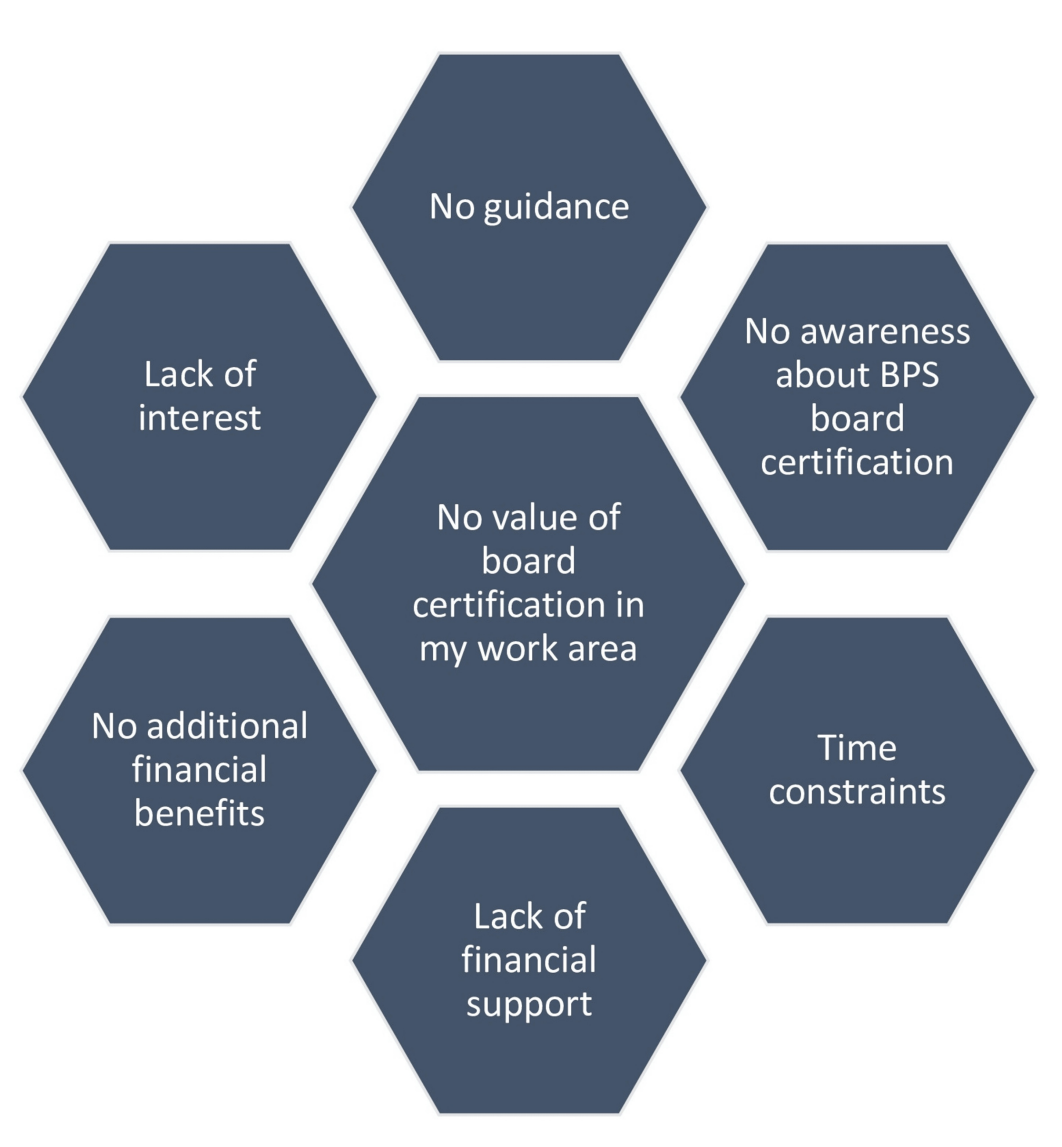
Barriers affecting pharmacist specialization This figure was created by the authors BPS: Board of Pharmacy Specialties

Of note, some clinical pharmacists may not be interested in specialization, particularly with the benefits thereof being very minimal. This might be because no specialty services are offered in their pharmacy settings, and hence they have no motivation to specialize. Alternatively, it is possible that their organization does not recognize the value of board certification, and hence they have minimal desire for certification. Figure 2 summarizes these barriers.

Challenges and opportunities related to fostering pharmacist board certification

A major issue noted regarding board certification as seen from a study in India is that the majority of the pharmacists are unaware of such specialization programs [18]. Critically, both senior and young graduates of clinical pharmacy should be well informed and made aware of important programs such as board certification. This will lead to an increase in the number of individuals being certified from India. At this point in the Indian Pharmacy Practice scenario, it’s difficult to determine the direct impact of pharmacist board certification because much more development is needed. However, proper sensitization and education of pharmacy professionals will ultimately result in an increase in the number of board-certified pharmacists in the years to come.

Moreover, it has been noted that there is minimal to no recognition of pharmacist board certification from respective employers and this has discouraged more clinical pharmacists from specializing. This however can be addressed by enlightening the hospital management more on the need for the specialization and firstly, it should be seen from the outstanding output of the specialized clinical pharmacist within the healthcare team. Importantly, financial constraints have been noted to be a major barrier in preventing the development of pharmacist specialization. We urge both employers and the respective bodies to give considerate financial support to their pharmacists interested in specializing since this will ultimately improve patient therapeutic care in a healthcare setting.

A study by Karattuthodi et al. showed that the knowledge and attitudes of Indian pharmacists towards BPS certification were less and it should be improved through the creation of awareness and motivating eligible pharmacists to do so. Their study revealed crucial information about the low knowledge of pharmacist specialization hence we have decided to write this perspective on the various ways to improve board certification not only among senior members but also postgraduate pharmacy students [19]. A review by Tiwari showed that there’s a need for more experience among the pharmacy practice faculty because the majority of them haven’t been to practical clinical settings hence they cannot guide properly their student’s in the need for board certification [10].

With the inception and graduation of more PharmD and M. Pharm Pharmacy Practice graduates in India, the clinical pharmacy profession must be elevated through special training. One of the best ways to achieve this is through creating awareness and encouraging more graduates to undergo the pharmacist board certification which ultimately does improve the pharmacist’s credibility within their area of practice.

India currently has only four board-certified pharmacists despite the large numbers of graduating students (8000 + PharmD) yearly [20]. This should be a wake-up call for all who are in the clinical pharmacy profession to give more priority to such kinds of certifications which has proved to be effective not only within the healthcare system but also the pharmacists' job satisfaction. Nevertheless, it is still important to recognize the challenges that most clinical pharmacists face regarding BPS.

## Conclusions

This review provides a comprehensive overview of the evolution of pharmacy practice globally and offers insightful comparisons between developed nations and India. We have compiled a significant amount of information on various aspects of pharmacy practice, including technological advancements, regulatory frameworks, and educational requirements across different countries. Our focus on the potential for growth in Indian pharmacy practice, particularly through specialization and board certification, is especially noteworthy. We highlight key challenges and opportunities in the field, which could serve as a useful starting point for further research and policy discussions aimed at advancing pharmacy practice in India.
